# Tryptophan-derived metabolites and BAK1 separately contribute to *Arabidopsis* postinvasive immunity against *Alternaria brassicicola*

**DOI:** 10.1038/s41598-020-79562-x

**Published:** 2021-01-15

**Authors:** Ayumi Kosaka, Marta Pastorczyk, Mariola Piślewska-Bednarek, Takumi Nishiuchi, Erika Ono, Haruka Suemoto, Atsushi Ishikawa, Henning Frerigmann, Masanori Kaido, Kazuyuki Mise, Paweł Bednarek, Yoshitaka Takano

**Affiliations:** 1 Laboratory of Plant Pathology, Graduate School of Agriculture, Kyoto University, Kyoto, 606-8502 Japan; 2 Institute of Bioorganic Chemistry, Polish Academy of Sciences, Noskowskiego 12/14, 61-704 Poznan, Poland; 3 Advanced Science Research Center, Kanazawa University, Kanazawa, Japan; 4 Department of Bioscience and Biotechnology, Fukui Prefectural University, Fukui, 910-1195 Japan; 5 Max Planck Institute for Plant Breeding Research, Carl-von-Linne-Weg 10, 50829 Cologne, Germany

**Keywords:** Microbiology, Molecular biology, Plant sciences

## Abstract

Nonhost resistance of *Arabidopsis thaliana* against the hemibiotrophic fungus *Colletotrichum tropicale* requires PEN2-dependent preinvasive resistance and CYP71A12 and CYP71A13-dependent postinvasive resistance, which both rely on tryptophan (Trp) metabolism. We here revealed that CYP71A12, CYP71A13 and PAD3 are critical for *Arabidopsis’* postinvasive basal resistance toward the necrotrophic *Alternaria brassicicola*. Consistent with this, gene expression and metabolite analyses suggested that the invasion by *A. brassicicola* triggered the CYP71A12-dependent production of indole-3-carboxylic acid derivatives and the PAD3 and CYP71A13-dependent production of camalexin. We next addressed the activation of the CYP71A12 and PAD3-dependent postinvasive resistance. We found that *bak1*-*5* mutation significantly reduced postinvasive resistance against *A*. *brassicicola*, indicating that pattern recognition contributes to activation of this second defense-layer. However, the *bak1*-*5* mutation had no detectable effects on the Trp-metabolism triggered by the fungal penetration. Together with this, further comparative gene expression analyses suggested that pathogen invasion in *Arabidopsis* activates (1) CYP71A12 and PAD3-related antifungal metabolism that is not hampered by *bak1*-*5*, and (2) a *bak1*-*5* sensitive immune pathway that activates the expression of antimicrobial proteins.

## Introduction

The resistance of an entire plant species against all isolates of particular pathogens is called nonhost resistance^1^. *Colletotrichum tropicale* (hereafter *Ctro*), formally called *C*. *gleosporioides*, is a hemibiotrophic fungal pathogen that causes anthracnose on its host mulberry; however, it is not able to enter the nonhost *Arabidopsis* because *Arabidopsis* blocks pathogen entry via activation of preinvasive resistance. Preinvasive resistance of *Arabidopsis* against *Ctro* involves *PENETRATION2* (*PEN2*) and *PEN3*^2–8^. Nonhost preinvasive resistance toward *Ctro* also needs *EDR1* (*ENHANCED DISEASE RESISTANCE 1*)^9^.

Importantly, even the *pen2 edr1* mutant is still not fully susceptible to *Ctro*^10^, because strong postinvasive resistance is newly activated once *Ctro* enters epidermal cells of the mutants defective in the preinvasive resistance. We reported previously that *Arabidopsis cyp79B2 cyp79B3* double mutant is fully susceptible to the nonadapted pathogen *Ctro*, i.e., the mutant is defective in both preinvasive and postinvasve resistance^10^. CYP79B2 and CYP79B3 are key enzymes for the biosynthesis of tryptophan (Trp)-derived antimicrobial metabolites. CYP79B2/CYP79B3 convert Trp into indole-3-acetaldoxime (IAOx)^11^, and this precursor is then converted into several compounds for antimicrobial immunity, such as PEN2 substrates indole-glucosinolates (IGs), PAD3 (PHYTOALEXIN-DEFICIENT3)-dependent camalexin, and 4-hydroxy-ICN (4-OH-ICN) whose biosynthesis requires CYP82C2^12–14^. We have shown that the *pen2 pad3* mutant is partially defective in postinvasive resistance to *Ctro*, indicating that the *Arabidopsis* phytoalexin, camalexin, is a critical factor for this.

In addition to serving as a precursor of IGs and camalexin, IAOx can be also converted to indole-3-carboxylic acid and its derivatives (ICAs). We have shown recently that CYP71A12 but not CYP71A13 has an important contribution to the accumulation of ICAs in leaves upon both *Ctro* and *P*. *cucumerina* inoculation^15^. On the other hand, loss of CYP71A13 reduced camalexin accumulation in leaves upon infection by multiple pathogens^15,16^, whereas a single loss of CYP71A12 did not reduce camalexin accumulation upon *Ctro* and *P*. *cucumerina* infection^15^. These findings suggest distinct roles of these two homologous P450 monooxygenases in the responses toward pathogen infection. Importantly, the *pen2 cyp71A12* double mutant exhibits a partial reduction in postinvasive resistance to *Ctro*^15^, which was similar to that of the *pen2 pad3* plants. This indicates that CYP71A12-dependent synthesis of ICAs as well as camalexin synthesis is critical for postinvasive resistance to *Ctro,* whereas CYP71A12 and PAD3 are dispensable for the preinvasive resistance: *Ctro* cannot invade the *pad3* single mutant or the *cyp71A12* single mutant^15^.

It has been reported that in addition to the above mentioned pests, camalexin is critical for the immunity of *Arabidopsis* to additional filamentous pathogens including a necrotrophic fungus *Alternaria brassicicola* (hereafter called *Ab*)^17,18^. However, it remains unclear whether CYP71A12 and ICAs are involved in *Arabidopsis* immunity to other fungi including *Ab.*

Immune responses in plants, including biosynthesis of specialized metabolites, can be triggered by pattern recognition-receptor (PRRs). BAK1 (BRASSINOSTEROID INSENSITIVE 1-ASSOCIATED RECEPTOR KINASE 1) is known to act as a coreceptor with multiple PRRs, including FLS2 and EFR, via ligand-induced heteromerization^19–22^. BAK1 was initially identified as a positive regulator of the brassinosteroid response (BR)^23,24^. Correspondingly, the *bak1* null mutants such as *bak1-4* mutant have a defect not only in FLS2 and EFR-dependent immune responses, but are also hyposensitive to BR. In contrast to the null alleles, the *bak1–5* allele is impaired in pathogen-associated molecular pattern (PAMP)-triggered immunity, but not in BR signaling^25^. Importantly, *bak1-5* is more severely impaired in defense responses than the null mutant *bak1-4*^25^.

Here, we investigated whether CYP71A12-dependent synthesis of ICAs might be involved in *Arabidopsis* immunity, especially postinvasive resistance, to *Ab* that infects Brassicaceae plants. As a result, we found that the invasion by *Ab* triggers the CYP71A12-dependent accumulation of ICAs as well as camalexin. Furthermore, evaluation of lesion development and microscopic observation of the pathogen invasion suggested the involvement of CYP71A12, together with PAD3, in postinvasive resistance to *Ab*.

We also asked how *Arabidopsis* recognizes *Ab* invasion to activate the CYP71A12 and PAD3-dependent postinvasive resistance. To this end we investigated function of BAK1 in immunity and ICAs formation and found that the two *bak1* mutations, especially *bak1–5*, reduce postinvasive resistance to *Ab*, revealing the involvement of a PRR system in the recognition of *Ab* invasion for the activation of defense. Unexpectedly, we found that the *bak1–5* mutation has no negative impact on the invasion-triggered activation of biosynthesis of camalexin or ICAs derived from Trp.

## Results

### CYP71A12 contributes to the immunity of *Arabidopsis* against the necrotrophic pathogen *Alternaria brassicicola* independently of CYP71A13 and PAD3

**Figure 1:**
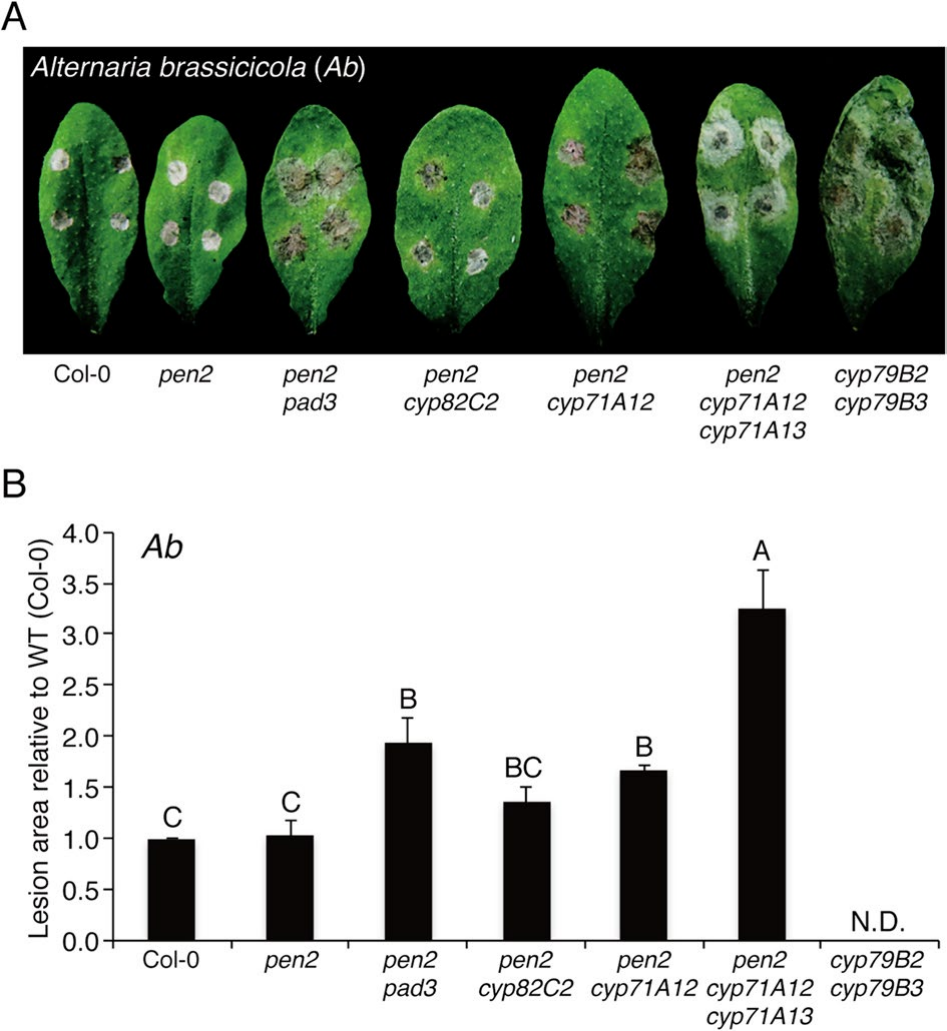
*PAD3* and *CYP71A12* are involved in the immunity of *Arabidopsis* against the necrotrophic pathogen *Alternaria brassicicola* (*Ab*). (**A**) Lesion development caused by *Ab* on *Arabidopsis* mutant plants with defects in Trp metabolism pathways. Conidial suspensions (1 × 10^5^ conidia/mL) of *Ab* were drop-inoculated onto mature leaves of 4–5-week-old plants. The photograph was taken at 4 days postinoculation (dpi). (**B**) Quantification of lesion development. Conidial suspensions of *Ab* were drop-inoculated onto tested plants. At 4 dpi, lesion areas were measured and the relative values to Col-0 (WT plants) were calculated. Means and standard deviations (SDs) were calculated from three independent experiments. The statistical significance of differences between means was determined by Tukey’s honestly significant difference (HSD) test. Means not sharing the same letter are significantly different (*P* < 0.05). N.D., not determined.

*Ab* is a necrotrophic fungal pathogen that infects several Brassicaceae spp., including cabbage and canola but is restricted within limited lesions when inoculated on leaves of *Arabidopsis* accession Col-0^9,17,26^. To investigate the roles of CYP71A12 in the postinvasive immunity of *Arabidopsis* against *Ab*, we used the Ryo-1 strain of *Ab* for the inoculation assay (Fig. 1). During our former study on postinvasive resistance against *Ctro* we investigated Trp metabolism-related mutants including *cyp71A12* in the *pen2* background, because *Ctro* was not able to invade these mutants in the absence of the *pen2* mutation (15). In this study, to focus on postinvasive resistance against *Ab,* we used these *pen2* background mutants for the *Ab* inoculation assay to exclude a potential effect of PEN2 on preinvasive resistance against this pathogen. Our evaluation of lesion development at 4 days postinoculation (dpi) revealed that the *pen2 cyp71A12* mutant showed enhanced susceptibility to *Ab,* as compared with the *pen2* plants, indicating contribution of CYP71A12 to the immunity towards *Ab* (Fig. 1). We also found that lesion development in the *pen2* mutant was not significantly different from that in the wild-type (WT) Col-0 leaves (Fig. 1), suggesting that opposite with the impact on several filamentous pathogens^2,6^, PEN2 has likely no detectable contribution to the *Arabidopsis* immunity against *Ab* at least in the Col-0 accession.

We also found that the *pen2 pad3* mutants have increased susceptibility to the *Ab* Ryo-1 (Fig. 1), indicating the importance of PAD3-dependent camalexin synthesis, consistent with previous reports^17,26^. Also, an additional mutation in *CYP71A13* further increased lesion development in *pen2 cyp71A12* (Fig. 1). Opposite with *PAD3* and *CYP71A12/A13*, *cyp82C2* mutation did not cause significant changes in *Ab* development suggesting that *CYP82C2* together with 4-OH-ICN do not contribute to the immunity toward this pathogen (Fig. 1). We were unable to perform proper quantitative analysis of lesion development in the *cyp79B2 cyp79B3* mutant at 4 dpi because the lesions had already merged. However, quantitative analysis at 3 dpi revealed that the *cyp79B2 cyp79B3* mutant was the most susceptible to *Ab* among all tested genotypes (Supplementary Fig. S1). As *PEN2* appeared to be unlikely essential for the immunity against *Ab*, we also investigated the effects of the mutations of *PAD3*, *CYP71A12*, *CYP71A13* and *CYP82C2* on the immunity to *Ab* in the WT background in addition to the *pen2* background by using *pad3*, *cyp82C2*, *cyp71A12*, *cyp71A12 cyp71A13* mutants. The obtained result was similar to the finding on the mutations in the *pen2* background (Supplementary Fig. S2).

### Biosynthesis of ICAs and camalexin occurs during postinvasive resistance toward *Ab*

Pastorczyk, M. et al.^15^ reported that (1) the expression of *CYP71A12* and *PAD3* is strongly induced in the *pen2* mutant defective in preinvasive resistance, but not in WT, upon the inoculation of the non-adapted pathogen *Ctro* and (2) *CYP71A12* and *PAD3* are involved in postinvasive resistance against *Ctro*. To assess whether *CYP71A12* and *PAD3* are expressed during postinvasive resistance against *Ab*, we investigated the expression pattern of these genes at several time points after *Ab* inoculation (at 4, 12, 24, and 48 h postinoculation, hpi). Although lesion development by *Ab* in *pen2* was comparable to that in WT (Fig. 1), we used the *pen2* mutant for the analysis to exclude a potential contribution of PEN2 to preinvasive resistance to *Ab.*

**Figure 2:**
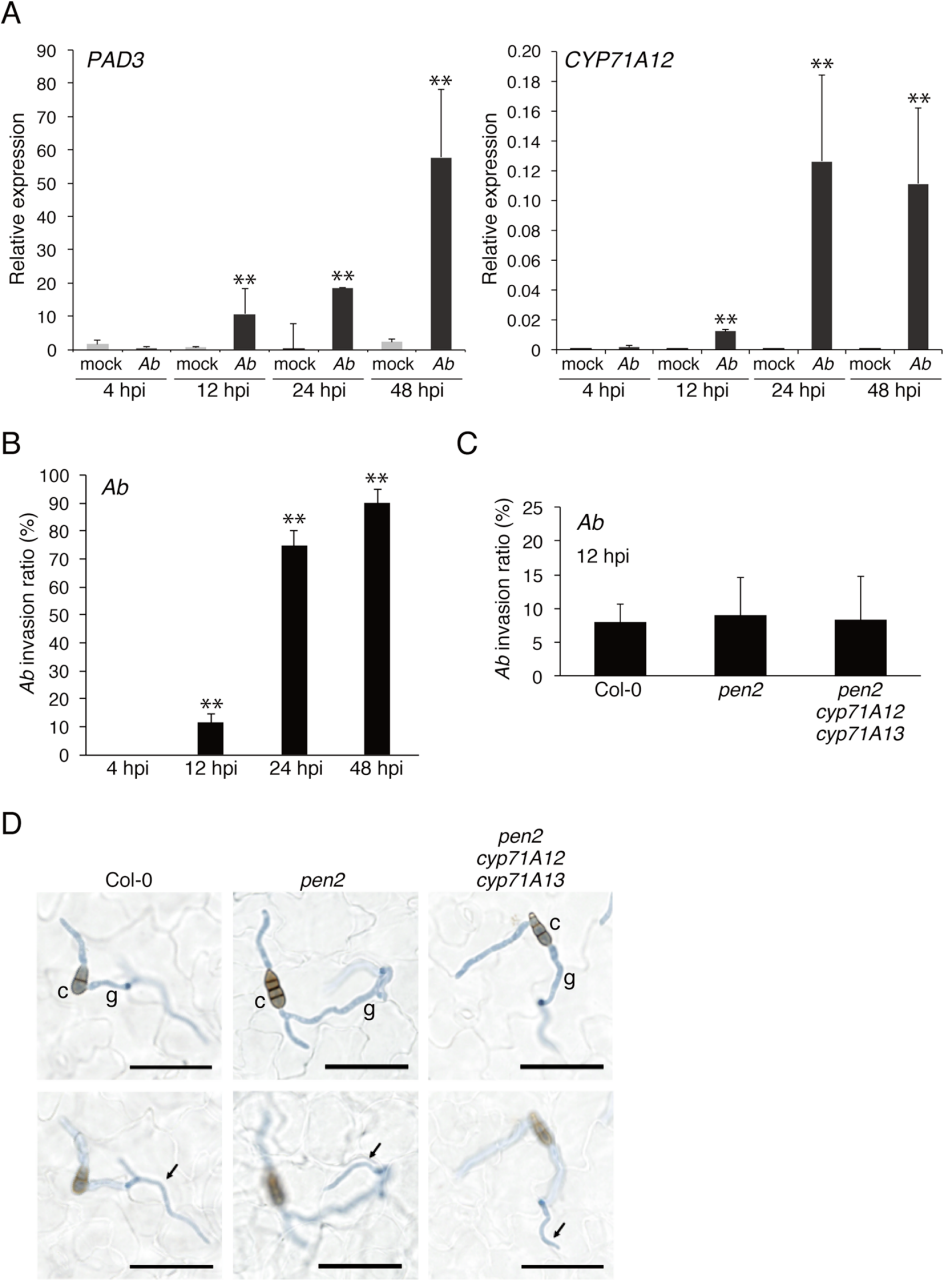
The invasion of *Ab* activates the expression of *PAD3* and *CYP71A12*. (**A**) Expression of *PAD3* and *CYP71A12* following *Ab* inoculation. Conidial suspensions (5 × 10^5^ conidia/mL) of *Ab* were spray-inoculated onto 4–5-week-old *pen2* plants and kept at 100% humidity. The samples were collected at 4, 12, 24, and 48 hpi. Each gene transcript was quantified by quantitative polymerase chain reaction (qPCR) using the gene-specific primers listed in Supplementary Table S2. Values were normalized to the expression level of *UBC21*. The means and SDs were calculated from three independent experiments. Statistical comparisons between mock and *Ab* treated samples were conducted using two-tailed Student’s *t* tests (**P < 0.01). (**B**) Quantitative analysis of the *Ab* invasion ratio among *pen2* plants. Conidial suspensions (1 × 10^5^ conidia/mL) of *Ab* were drop-inoculated onto *pen2* plants and kept at 100% humidity. The inoculated leaves were collected at 4, 12, 24, and 48 hpi, and then subjected to a trypan blue viability staining assay. The presence or absence of invasive hyphae from at least 50 germinating conidia were counted in each experiment. The means and SDs were calculated from three independent experiments. Statistical comparisons of the *Ab* invasion ratios at 12, 24, and 48 hpi against that at 4 hpi were conducted using two-tailed Student’s *t* tests (**P < 0.01). (**C**) *PEN2*, *CYP71A12*, and *CYP71A13* are dispensable for preinvasive resistance against *Ab*. Aliquots of 5 μL of *Ab* conidial suspension were drop-inoculated onto leaves of 4–5-week-old plants. At 12 hpi, the inoculated leaves were collected and stained with trypan blue, and then invasive hyphae were observed by light microscopy. At least 50 germinating conidia were counted in each experiment. The means and SDs were calculated from three independent experiments. Statistical analysis using two-tailed Student’s *t* tests showed no significant differences among the genotypes. (**D**) Light microscopy observations. At 12 hpi, a part of the germinating conidia developed invasive hyphae (arrows) inside the inoculated plants. The top images are focused on conidium and the bottom images are focused on invasive hypha. *c* conidium, *g* germ tube. Bars = 50 µm.

We found that the expression of *CYP71A12* and *PAD3* started to be induced at 12 hpi in *pen2*, and the corresponding expression levels were stronger elevated at later time points (Fig. 2A). By contrast, we did not detect any induction at 4 hpi (Fig. 2A). In parallel, we also investigated the temporal infection behavior of *Ab* in *Arabidopsis*. We found that conidia of *Ab* had already germinated at 4 hpi, however, we did not detect any host invasion at this time (Fig. 2B). The fungus started to invade the *pen2* plants at 12 hpi and the invasion ratio became elevated at later time points (Fig. 2B,D). These findings indicate a link between *CYP71A12* and *PAD3* induction and the initiation of host invasion in the *Ab*-*Arabidopsis* interactions, strongly suggesting that the expressions of *CYP71A12* and *PAD3* are triggered by *Ab* invasion. Furthermore, we also revealed that simultaneous loss of both *CYP71A12* and *CYP71A13* produced no detectable effects on the preinvasive resistance against *Ab* (Fig. 2C,D). These findings suggest that *CYP71A12* and *CYP71A13* are involved in the postinvasive resistance against *Ab*. We also found that the invasion ratio of *Ab* in *pen2* was not significantly different from that in WT (Fig. 2C), in contrast to the case of *Ctro*^6^.

**Figure 3:**
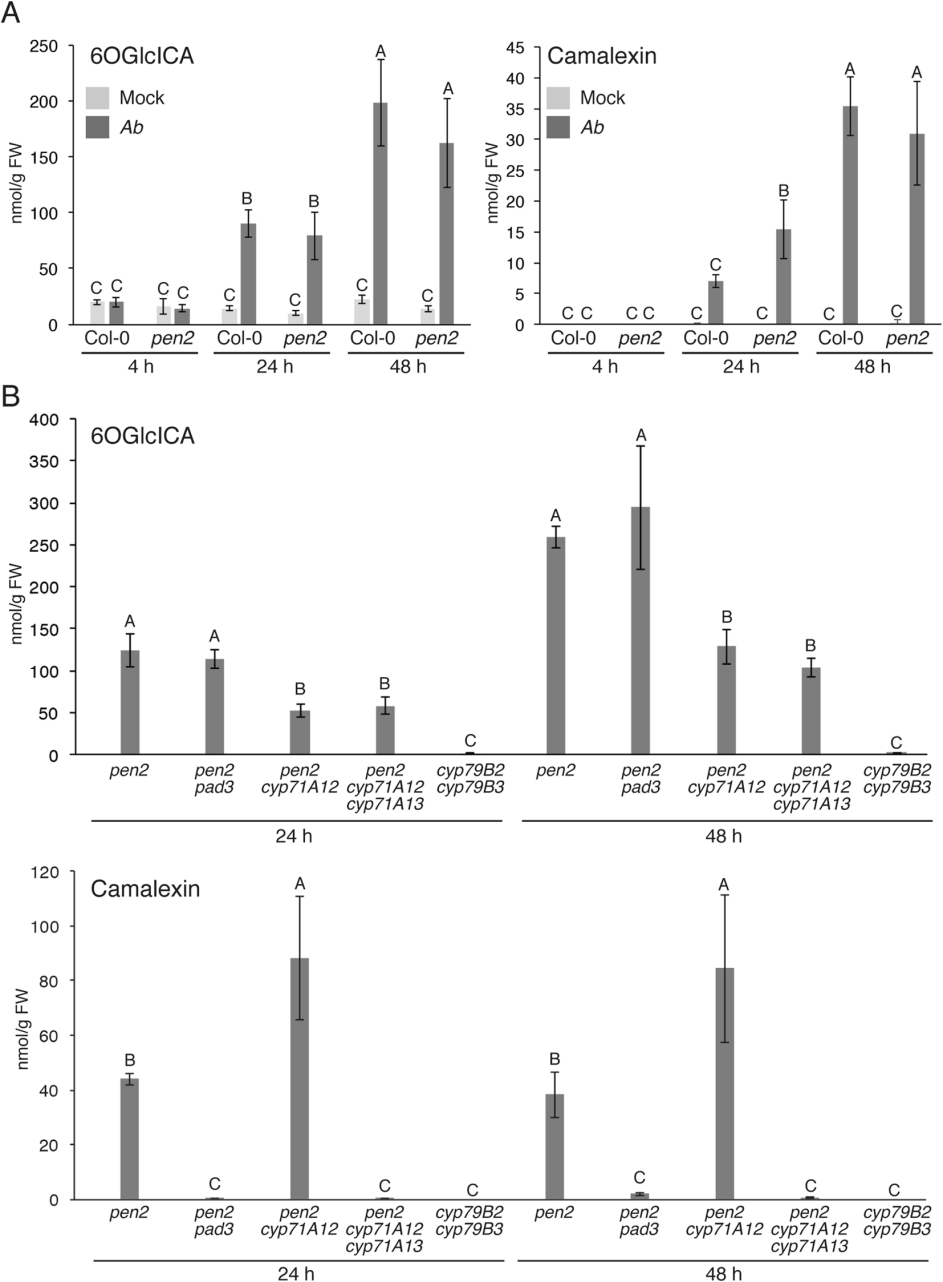
Invasion by *Ab* induced CYP71A12-dependent biosynthesis of 6-hydroxy-ICA and PAD3-dependent biosynthesis of camalexin. (**A**) The accumulation of 6-hydroxy-ICA (6OGlcICA) and camalexin in *Arabidopsis* plants inoculated with *Ab*. Conidial suspensions (5 × 10^5^ conidia/mL) of *Ab* were spray-inoculated onto Col-0 (WT) and *pen2* plants. As a control, water was sprayed as a mock treatment. The means of metabolites (nmol/g fresh weight, FW) and SDs from four biological independent samples are shown in the graph. The statistical significance of differences between means was determined by Tukey’s HSD test. Means not sharing the same letter are significantly different (*P* < 0.05). (**B**) *CYP71A12* is required for the *Ab*-triggered accumulation of 6OGlcICA, whereas *PAD3* and *CYP71A13* are required for the accumulation of camalexin. Conidial suspensions (5 × 10^5^ conidia/mL) of *Ab* were spray-inoculated onto tested mutant lines. The statistical significance of differences between means in each time point was determined by Tukey’s HSD test. Means not sharing the same letter are significantly different (*P* < 0.05).

We then assessed whether our observations made at the gene expression level could be supported with metabolite profiles. We found that the pathogen-induced accumulation of two ICAs, glucoside of 6-hydroxy-indole-3-carboxylic acid (6OGlcICA) and glucose ester of indole-3-carboxylic acid (ICAGlc), was detected at 24 hpi, but not at 4 hpi with *Ab* in both WT and *pen2* (Fig. 3A; Supplementary Fig. S3), which matched strongly with the gene expression data (Fig. 2A). Similar results were also obtained during the analysis of camalexin accumulation (Fig. 3A). Therefore, we conclude that the synthesis of ICAs and camalexin is triggered by *Ab* invasion.

### Reduced accumulation of ICAs correlates with the breakdown of the postinvasive resistance toward *Ab*

We have reported recently that the accumulation of ICAs triggered by inoculation with *P*. *cucumerina* is reduced in the *cyp71A12*, but not in the *cyp71A13* mutant^15^. By contrast, the *P*. *cucumerina*-triggered accumulation of camalexin was reduced in the *cyp71A13*, but not in the single *cyp71A12* mutant^15^. Therefore, we assumed that the CYP71A12-dependent production of ICAs contributes to *Arabidopsis* postinvasive immunity to *Ab* independently of CYP71A13-dependent camalexin production. As mentioned above, we also found that the *cyp79B2 cyp79B3* mutant exhibited more severe defects in postinvasive resistance against *Ab* than the *pen2 cyp71A12 cyp71A13* mutant (Fig. 1 and Supplementary Fig. S1), although the reason was not clear. To get further insights on these aspects, we investigated the Trp-related metabolite profiles of the aforementioned mutants: *pen2*, *pen2 pad3*, *pen2 cyp71A12*, *pen2 cyp71A12 cyp71A13*, and *cyp79B2 cyp79B3*.

Obtained results revealed that the simultaneous loss of *CYP79B2* and *CYP79B3* completely abolished the *Ab*-triggered accumulation of ICAs and camalexin, whereas the loss of *PAD3* canceled camalexin accumulation, but had no effects on the accumulation of ICAs (Fig. 3B; Supplementary Fig. S4). In the *pen2 cyp71A12* mutant, the *Ab*-triggered accumulation of ICAs was reduced significantly compared with the *pen2* mutant, opposite with camalexin accumulation that was rather increased compared with *pen2* (Fig. 3B; Supplementary Fig. S4). These results further strengthen the idea that the accumulation of ICAs triggered by the *Ab* invasion is critical for the postinvasive resistance of *Arabidopsis* against this necrotrophic fungal pathogen. In the *pen2 cyp71A12 cyp71A13* mutant, the ICA levels that accumulated upon *Ab* invasion were similar to those observed in the *pen2 cyp71A12* mutant, but camalexin accumulation in the triple mutant was completely diminished, in contrast to *pen2 cyp71A12* (Fig. 3B; Supplementary Fig. S4), further supporting the importance of CYP71A13 for the pathogen-induced accumulation of camalexin, but not ICAs.

It is noteworthy that the leaves of *pen2 cyp71A12 cyp71A13* mutant still accumulated clearly detectable amounts of ICAs at both 24 hpi and 48 hpi of *Ab*, whereas camalexin in this triple mutant was under the detection limit at the same time points (Fig. 3B; Supplementary Fig. S4). As mentioned above, the *cyp79B2 cyp79B3* mutant exhibited a more severe phenotype to *Ab* inoculation compared with the *pen2 cyp71A12 cyp71A13* mutant (Fig. 1A,B; Supplementary Fig. S1). The *cyp79B2 cyp79B3* mutant is entirely defective in the production of not only camalexin, but also ICAs (Fig. 3B; Supplementary Fig. S4). Also, both *cyp79B2 cyp79B3* and *pen2 cyp71A12 cyp71A13* mutants are commonly defective in biosynthesis of the PEN2-dependent IG-hydrolysis products (leading to unidentified antifungal compounds), these data suggested that the different levels of susceptibility to *Ab* between *pen2 cyp71A12 cyp71A13* and *cyp79B2 cyp79B3* is likely caused by the differential accumulation of ICAs in these two mutants.

In addition to ICAs and camalexin, CYP79B2/CYP89B3 enzymes are essential for biosynthesis of other Trp-derived metabolites including IGs^15,27^. The *pen2 cyp71A12 cyp71A13* mutant lacks the PEN2-dependent IG-hydrolysis products, but retains the ability to produce IGs, which can be activated by another enzyme. It has been reported that IG biosynthesis in *Arabidopsis* is controlled by the transcription factors MYB34, MYB51, and MYB122, whereas these factors are dispensable for the pathogen triggered biosynthesis of camalexin and ICAs^27^. Thus, to assess the possibility that the different susceptibility to *Ab* between *pen2 cyp71A12 cyp71A13* and *cyp79B2 cyp79B3* mutants might be linked to IG biosynthesis, we compared phenotypes of *pen2 cyp71A12 cyp71A13* and *cyp71A12 cyp71A13 myb34 myb51 myb122* lines following *Ab* inoculation. We first performed the metabolite analyses on the *cyp71A12 cyp71A13 myb34 myb51 myb122* mutant leaves at 24 hpi of *Ab* (Supplementary Fig. S5)*.* The obtained results showed that this mutant was defective in IG biosynthesis upon *Ab* infection. Subsequently, we tested susceptibility of this mutant towards *Ab* in an inoculation assay. We found that lesion development in the *cyp71A12 cyp71A13 myb34 myb51 myb122* mutant leaves was similar to that in the *pen2 cyp71A12 cyp71A13* mutant (Supplementary Fig. S6). These results indicate that PEN2-independent IG hydrolysis is likely not involved in the postinvasive resistance against *Ab*. Thus, the different susceptibility between the *pen2 cyp71A12 cyp71A13* mutant and the *cyp79B2 cyp79B3* mutant is not linked to IG-deficiency. Together with the involvement of CYP71A12-dependent ICAs synthesis in the postinvasive resistance of *Arabidopsis* against *Ab*, we consider that the remaining ICAs in *pen2 cyp71A12 cyp71A13* are still effective against *Ab.* Consistent with this idea, the *pen2 cyp71A12* mutant was more susceptible to *Ab* than the *pen2* mutant (Fig. 1).

### The *bak1–5* mutation reduces the postinvasive resistance of *Arabidopsis* to *Ab*, independently of pathogen-triggered ICAs and camalexin biosynthesis

Next, we asked how *Arabidopsis* recognizes the invasion by *Ab* to activate the biosynthesis of ICAs and camalexin. The candidate mechanism critical for this process is the PRRs-dependent PAMP recognition machinery^28,29^. When *Arabidopsis* recognizes PAMPs, at least some of the cognate PRRs, including FLS2 and EFR receptor-like kinases (RLKs), form complexes with coreceptors such as BAK1^19–22^. Therefore, we decided to assess the possible involvement of BAK1 in the invasion-triggered accumulation of ICAs and camalexin. For this purpose, we used two mutant alleles of *BAK1*, *bak1–4* and *bak1–5*; of these, *bak1–4* is a *BAK1* null allele^19^, and *bak1–5* is a semi-dominant allele with a specific phenotype related to PAMP responsiveness^25^. Importantly, it was reported that *bak1-5* is more severely impaired in elf18 and flg22 responses than the null mutant *bak1-4*^25^.

**Figure 4:**
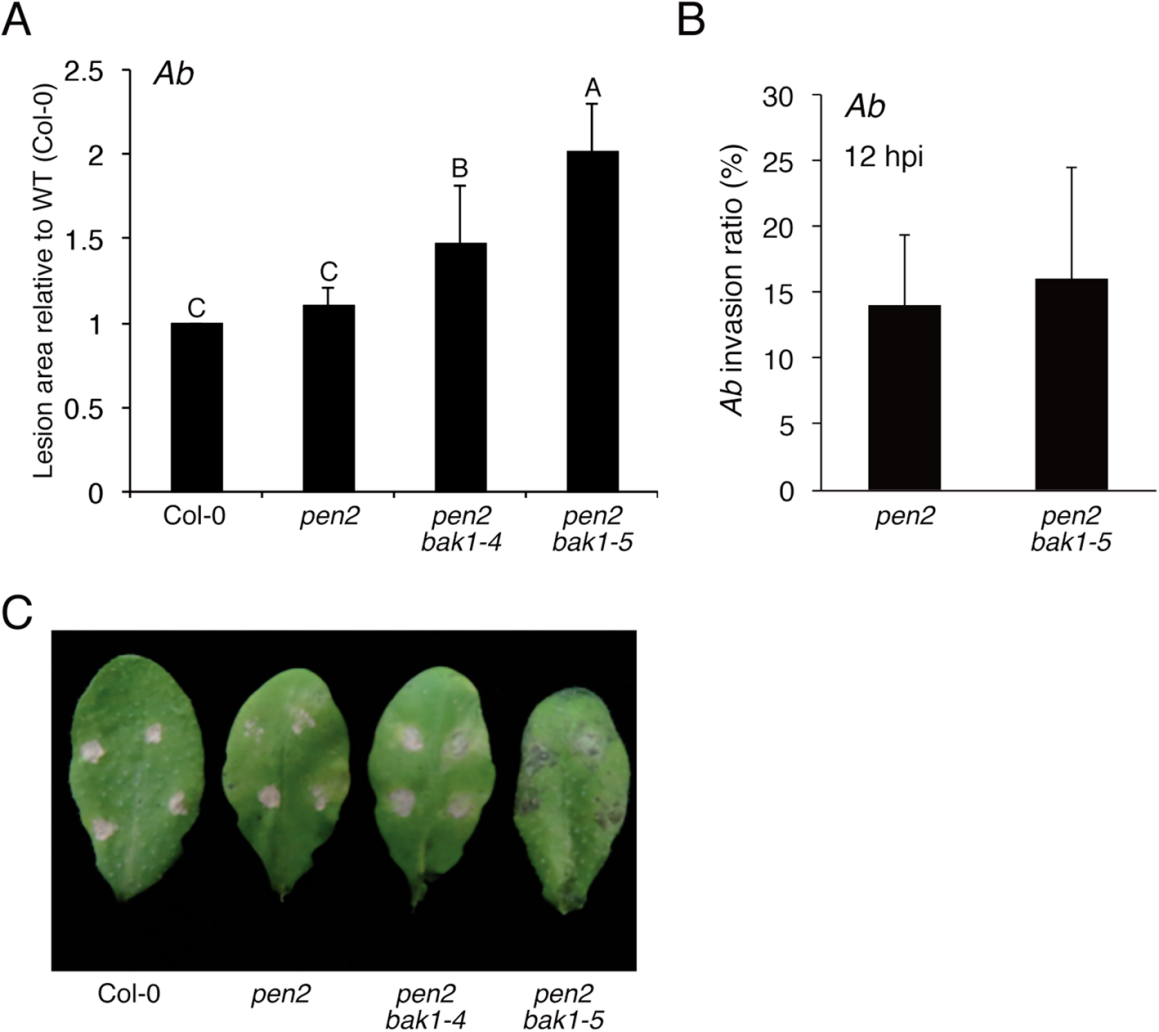
The *bak1* mutations reduced the immunity of *Arabidopsis* against *Ab*. (**A**) Quantification of lesion development. Conidial suspensions of *Ab* (1 × 10^5^ conidia/mL) were drop-inoculated onto true leaves of 4–5-week-old plants. At 4 dpi, lesion areas were measured and the relative values to Col-0 (WT plants) were calculated. The means and SDs were calculated from three independent experiments. The statistical significance of differences between means was determined by Tukey’s HSD test. Means not sharing the same letter are significantly different (*P* < 0.05). (**B**) The *bak1–5* mutation did not reduce preinvasive resistance against *Ab* in the *pen2* mutant. Aliquots of 5 μL of conidial suspension (1 × 10^5^ conidia/mL) of *Ab* were drop-inoculated onto leaves of 4–5-week-old plants of the tested mutants. At 12 hpi, the inoculated leaves were collected and stained with trypan blue, and then invasive hyphae were observed under light microscopy. At least 50 germinating conidia were counted in each experiment. The means and SDs were calculated from three independent experiments. Statistical analysis using two-tailed Student’s *t* tests showed no significant differences between *pen2* and *pen2 bak1–5* mutants. (**C**) Lesion development caused by *Ab* on two *pen2 bak1* mutants. Conidial suspensions (1 × 10^5^ conidia/mL) of *Ab* were drop-inoculated onto mature leaves of 4–5-week-old plants. The photograph was taken at 4 dpi.

Because the main results on gene expression and metabolite accumulation were derived from the *pen2*-background mutants in this study, we used the *pen2 bak1–4* mutant^30^ and the newly generated *pen2 bak1–5* mutant. We first performed *Ab* inoculation assay on these plant lines and found that both *pen2 bak1–4* and *pen2 bak1–5* plants have reduced immunity to *Ab*, although the *pen2 bak1–5* plants were more susceptible than the *pen2 bak1–4* plants (Fig. 4A,C). Similar results were observed for single *bak1–4* and *bak1–5* as compared with WT plants (Supplementary Fig. S7). We then evaluated whether the *bak1–5* mutation would reduce preinvasive resistance to *Ab*. To assess this, we compared the invasion behavior of *Ab* in the *pen2* mutant with that in the *pen2 bak1–5* mutant. The invasion ratio in the *pen2 bak1–5* mutant was similar to that in the *pen2* mutant, suggesting that the *bak1–5* mutation does not have detectable impact on preinvasive resistance to *Ab* (Fig. 4B). These results indicate that the *bak1–5* mutation reduces postinvasive resistance to *Ab*, i.e., PRR systems likely function in the recognition of *Ab* invasion.

**Figure 5:**
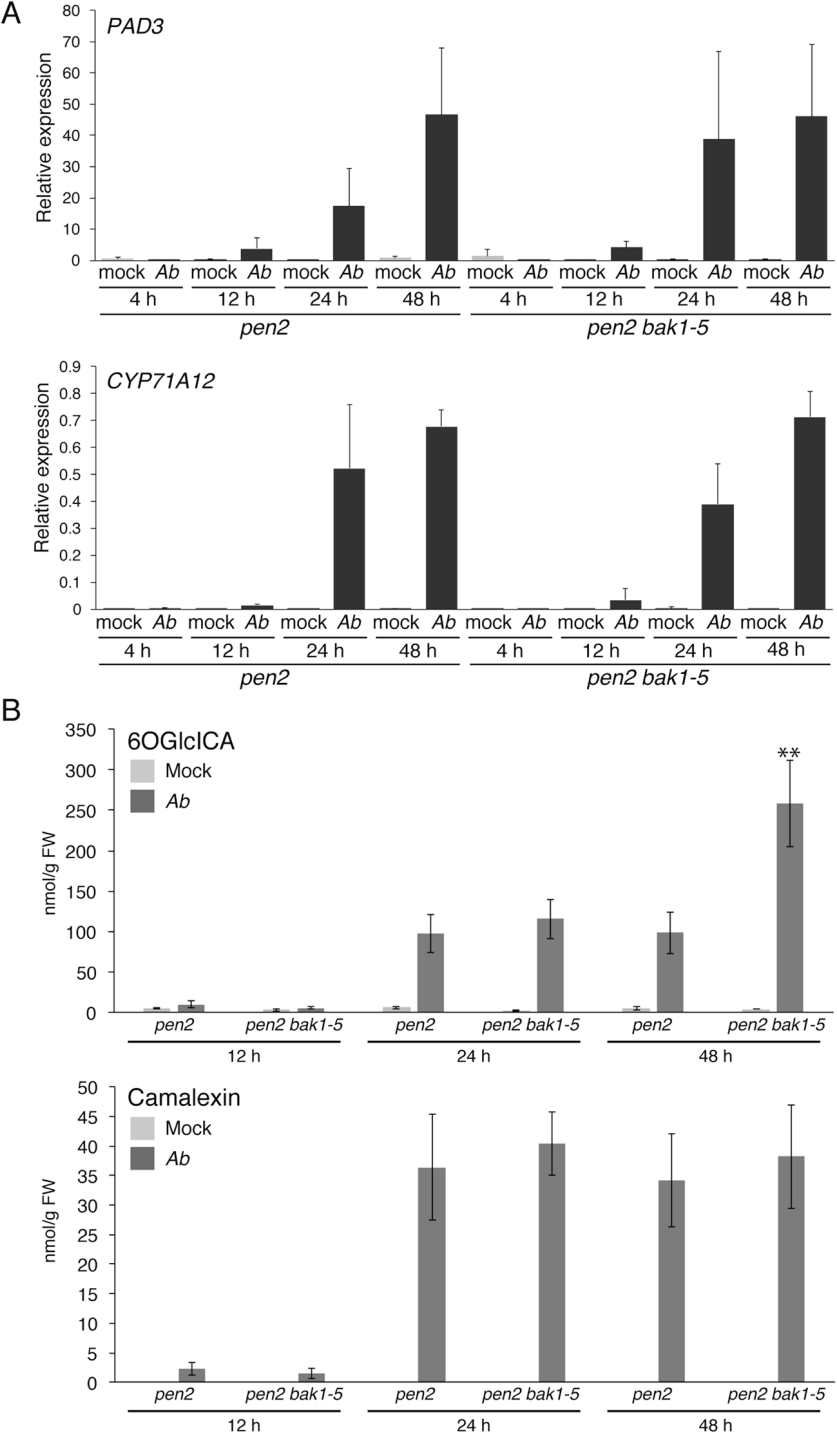
The *bak1–5* mutation did not reduce the *Ab*-invasion triggered accumulation of 6-hydroxy-ICA and camalexin. (**A**) The *Ab*-invasion triggered activation of *CYP71A12* and *PAD3* expression in the *pen2 bak1–5* mutant. Conidial suspensions (5 × 10^5^ conidia/mL) of *Ab* were spray-inoculated onto 4–5-week-old plants. The samples were collected at 4, 12, 24, and 48 hpi. Each gene transcript was quantified by RT–qPCR using the gene-specific primers listed in Supplementary Table S2. Values were normalized to the expression level of *UBC21*. The statistical comparison between *Ab*-treated *pen2* and *Ab*-treated *pen2 bak1-5* at same time point samples was conducted using two-tailed Student’s *t* tests and did not show significant differences. (**B**) The *Ab* invasion-triggered accumulations of 6-hydroxy-ICA (6OGlcICA) and camalexin were not canceled by the *bak1–5* mutation. Conidial suspensions (5 × 10^5^ conidia/mL) of *Ab* were spray-inoculated onto the tested mutant lines. The means of metabolites (nmol/g FW) and SDs from four biological independent samples are shown in the graph. The statistical comparison between *pen2* and *pen2 bak1-5* at same timepoint was conducted using two-tailed Student’s *t* tests (**P < 0.01).

Because we found that the *bak1–5* mutation reduced postinvasive resistance to *Ab*, we next investigated whether *bak1–5* would have negative effects on the *Ab* invasion-triggered activation of camalexin and ICAs biosynthesis. We checked the gene expression of *PAD3* and *CYP71A12* in the *pen2* and *pen2 bak1–5* mutants after *Ab* inoculation. Surprisingly, the *bak1–5* mutation did not cancel the induced expression of *PAD3* and *CYP71A12* in *pen2* (Figs. 2A, 5A). Furthermore, we found that the accumulation of camalexin and ICAs upon *Ab* invasion was not reduced in the *pen2 bak1–5* mutant compared with the *pen2* mutant (Fig. 5B and Supplementary Fig. S8). The accumulation level of ICAs was even higher in *pen2 bak1–5* than *pen2* at 48 hpi, which might be due to enhanced infection in *pen2 bak1–5* (Fig. 5B and Supplementary Fig. S8). Collectively, these results indicate that the *bak1–5* mutation does not reduce the *Ab*-triggered activation of camalexin and ICAs biosynthesis, although this mutation reduces postinvasive resistance to *Ab*.

### The *bak1–5* mutation reduces the *Ab* invasion-triggered expression of defense-related genes, including *GLIP1*.

**Figure 6:**
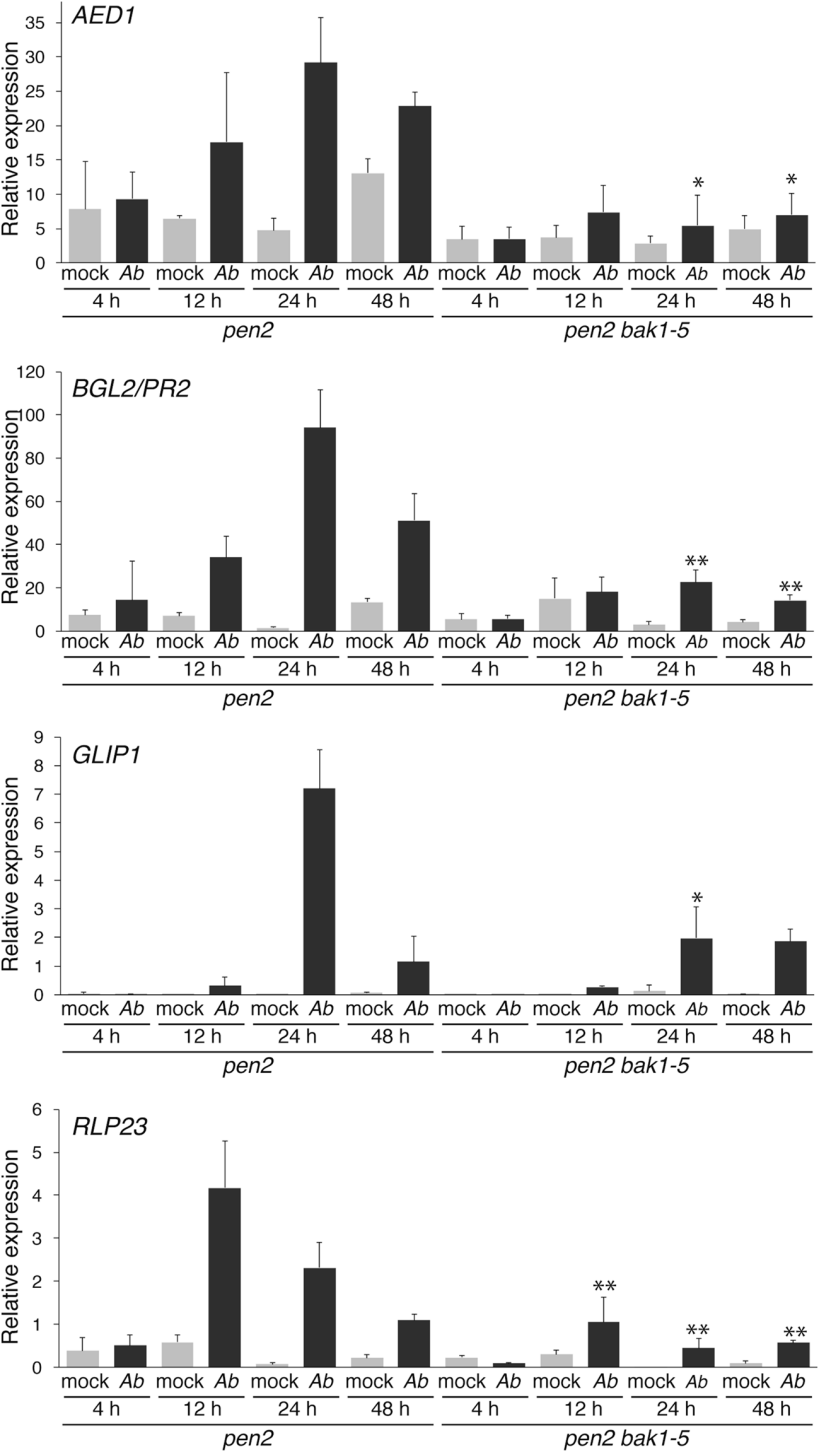
The *Ab* invasion-triggered expression levels of *AED1*, *BGL2/PR2*, *GLIP1*, and *RLP23* were reduced by the *bak1–5* mutation. Conidial suspensions (5 × 10^5^ conidia/mL) of *Ab* were spray-inoculated onto 4–5-week-old *pen2* and *pen2 bak1–5* plants, and then kept at 100% humidity. The samples were collected at 4, 12, 24, and 48 hpi. Each gene transcript was quantified by RT–qPCR using the gene-specific primers listed in Supplementary Table S2. Values were normalized to the expression level of *UBC21*. The means and SDs were calculated from three independent experiments. The statistical analysis was conducted by a two-tailed *t* test. The expression levels of each gene between *pen2* and *pen2 bak1–5* were compared at the same time points and treatment (*P < 0.05; **P < 0.01).

We further investigated the *bak1–5* sensitive pathways for postinvasive resistance against *Ab*. We performed comparative expression profiling experiments in *pen2* and *pen2 bak1–5* plants following *Ab* invasion using microarray analysis. *Ab* was inoculated to each plant, and RNA isolated from inoculated leaves at 24 hpi was subjected to microarray analysis. We focused on differentially expressed genes associated with the immune response based on Gene Ontology (GO) data (GO term 0006955: http://www.informatics.jax.org). As a result, we found that 14 genes had greater than a 2.5-fold change in expression. Interestingly, 11 were down-regulated, and three were up-regulated (Supplementary Table S1), implying that, compared with the *pen2* plants, the *pen2 bak1–5* plants exhibited a trend for reduced immune responses upon *Ab* invasion. Among the 11 down-regulated genes, four were shown previously to be involved in the *Arabidopsis* immune system using functional analyses such as the analysis of corresponding knockout mutants, including *AED1* (*APOPLASTIC, EDS1-DEPENDENT 1*)^31^, *BGL2*/*PR2*^32^, *GLIP1*^33,34^, and *RLP2*3 (*RECEPTOR-LIKE PROTEIN 23*)^35^. Notably, the *glip1* plants were reported to be more susceptible to *Ab* than the WT plant^33^. Subsequently, we performed reverse transcription quantitative polymerase chain reaction (RT–qPCR) analyses to investigate the expression levels of these four genes (*AED1*, *BGL2*/*PR2*, *GLIP1*, and *RLP23*) at 4, 12, 24, and 48 hpi in *pen2* and *pen2 bak1–5* plants inoculated with *Ab* (Fig. 6). As a control, we also investigated gene expression in the mock-treated plants. We confirmed that these four genes exhibited lower expression in the *pen2 bak1–5* than in the *pen2* mutant at 24 hpi, consistent with the array data. Furthermore, the RT–qPCR analysis revealed that the expression of *AED1*, *BGL2*/*PR2*, *GLIP1*, and *RLP23* was lower at 4 hpi than at 12, 24, and 48 hpi (Fig. 6). Together with our finding that *Ab* did not invade at 4 hpi, but started to invade at 12 hpi (Fig. 2B), our results indicate that these genes are induced upon *Ab* invasion to function in postinvasive resistance. Such induced expression was continuously suppressed in the *pen2 bak1–5* plants (Fig. 6), further suggesting that this invasion-triggered expression depends on putative PRRs with function impaired in the *bak1–5* mutant. It is also noteworthy that the invasion-triggered expressions of *AED1*, *BGL2*/*PR2*, *GLIP1*, and *RLP23* were time-dependent, i.e., induced expression started to be down-regulated at 12 hpi (*RLP23*) or 24 hpi (*AED1*, *BGL2*/*PR2*, *GLIP1*) (Fig. 6), which is in contrast to the invasion-triggered expressions of *PAD3* and *CYP71A12*, which exhibited sustained elevations for up to 48 hpi (Figs. 2A, 5A).

## Discussion

We recently reported that CYP71A12 is indispensable for postinvasive resistance to the hemibiotrophic pathogen *Ctro* that is not adapted to *Arabidopsis*^15^. However, it remained obscure whether CYP71A12 is also involved in this invasion-triggered resistance of *Arabidopsis* against other fungal pathogens.

Here we found that the absence of functional CYP71A12 enhanced lesion development during colonization of *Arabidopsis* leaves with the necrotrophic pathogen *Ab*, indicating that this enzyme is required for the immune response of *Arabidopsis* against this fungus. *CYP71A12* was not induced at 4 hpi with *Ab* when the pathogen had not yet invaded, but it started to be induced upon *Ab* invasion (Fig. 2A). We also found that *CYP71A12* was dispensable for preinvasive resistance against *Ab* (Fig. 2C). Collectively, these results demonstrate that *CYP71A12* is required for postinvasive resistance against *Ab* as well as *Ctro*. Metabolic analyses showed that the enhanced accumulation of ICAs triggered by *Ab* invasion was reduced in the absence of functional CYP71A12. Thus, this enzyme is involved in the accumulation of ICAs following *Ab* invasion (Fig. 3B). Therefore, we consider that the CYP71A12-dependent synthesis of ICA and/or its derivatives upon *Ab* invasion is critical for postinvasive resistance to this fungus.

We also showed that CYP71A13 contributes to the postinvasive resistance against *Ab* via synthesis of camalexin, but not of ICAs (Fig. 3B). The result suggests the importance of camalexin for this second layer of defense, which is further supported by our phenotypic analyses of mutants defective in *PAD3* (Fig. 1 and Supplementary Fig. S2).

PEN2 is involved in preinvasive resistance against *Ctro*^6^, but we here revealed that it is dispensable for preinvasive resistance against *Ab* (Fig. 2C,D). Furthermore, PEN1 is known to be required for preinvasive resistance against nonadapted powdery mildews^36^, but dispensable for the *Colletotrichum* fungi and nonadapted *Alternaria alternata*^8,37^. It is also noteworthy that PEN2 is involved in preinvasive resistance against *A. alternata*, in contrast with *Ab*^37^. Thus, the molecular components that underlie preinvasive resistance vary between fungal pathogens, probably because pathogens have evolved various strategies for plant entry. By contrast, once these pathogens enter *Arabidopsis*, they commonly develop invasive structures inside the plants; thus, the hosts might deploy an universal defense systems to terminate further fungal growth.

We found that the *cyp79B2 cyp79B3* mutant is more susceptible to *Ab* than is the *pen2 cyp71A12 cyp71A13* mutant (Fig. 1 and Supplementary Fig. S1). Interestingly, this phenomenon is also observed in the infection by *Ctro* and *P*. *cucumerina*^15^. Metabolite analyses of plants upon *Ab* invasion revealed that camalexin accumulation in the *pen2 cyp71A12 cyp71A13* plants was almost the same as that in the *cyp79B2 cyp79B3* plants (Fig. 3B). Importantly, the *pen2 cyp71A1 cyp71A13* plants reduced their accumulation of ICAs, but still produced them to some degree, whereas the *cyp79B2 cyp79B3* plants were entirely defective in this regard (Fig. 3B, Supplementary Fig. S4). Consistent with this finding, several independent metabolic branches are supposed to contribute to endogenous ICAs levels, including contribution of Arabidopsis aldehyde oxidase 1 and CYP71B6^15,38,39^.

We also compared the phenotype of *pen2 cyp71A12 cyp71A13* mutants with that of *myb34 myb51 myb122 cyp71A12 cyp71A13* following *Ab* invasion and found no detectable differences in either mutants in terms of postinvasive resistance against *Ab*, indicating that the difference between *pen2 cyp71A12 cyp71A13* and *cyp79B2 cyp79B3* mutants is not caused by PEN2-unrelated IG metabolism products (Supplementary Figs. S5, S6). Collectively, these findings suggest that the lower susceptibility in the former mutants is caused by residual ICAs, i.e., ICAs contribute to postinvasive resistance in a dose-dependent manner. This supports the idea that ICAs or their derivatives work as antifungal compounds as opposed to functioning as signaling molecules for plant immune responses. However, we cannot exclude a possibility that accumulation of so far not-reported IAOx-derivatives whose biosynthesis is not dependent on CYP71A12 and CYP71A13 contribute to the difference observed in susceptibility of *pen2 cyp71A12 cyp71A13* and *cyp79B2 cyp79B3* plants.

We also investigated how *Arabidopsis* recognizes the invasion of fungal pathogens and then mounts its postinvasive resistance. We found that the two *bak1* mutations, especially the *bak1–5* mutation, reduce postinvasive resistance against *Ab* (Fig. 4). The finding that the negative effects of *bak1-5* on the postinvasive resistance towards *Ab* was higher than that of *bak1-4* is likely consistent with the previous works^25^.

**Figure 7:**
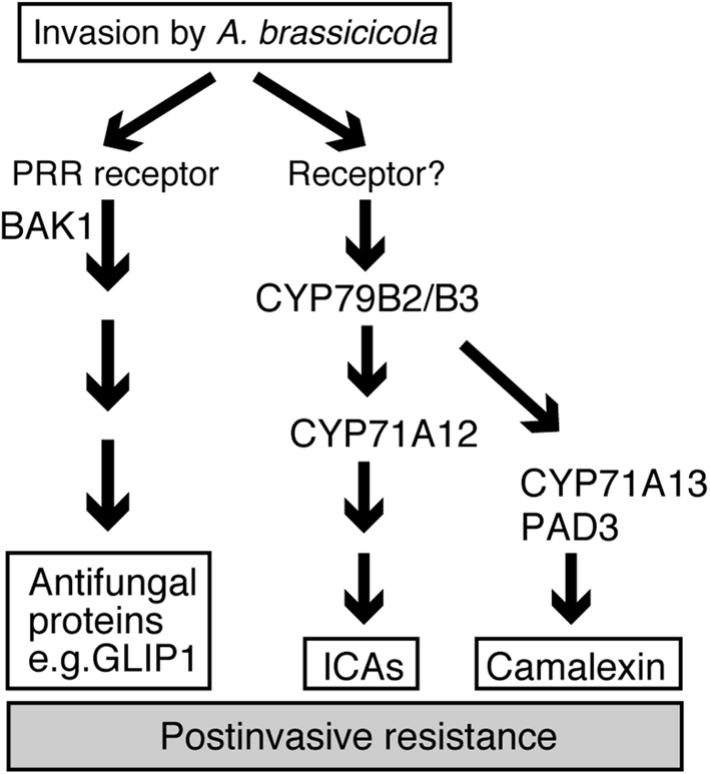
Summarized model for *Arabidopsis* postinvasive resistance against *Ab*. The CYP71A12-dependent production of ICAs is required for postinvasive resistance against *A. brassicicola*. The CYP71A13- and PAD3-dependent production of camalexin is also required for postinvasive resistance against *Ab*. The *bak1* mutations (especially *bak1-5*) reduced the postinvasive resistance, however, invasion-triggered activation of these Trp-related pathways is not canceled by *bak1-5*. The *bak1-5* sensitive pathway control the expression of antifungal protein genes (e.g. *GLIP1*).

Surprisingly, we found that the *bak1–5* mutation did not hamper the invasion-triggered expressions of *PAD3* and *CYP71A12* and the subsequent accumulation of ICAs and camalexin (Fig. 5). Thus, we postulate existence of another defense mechanism that is affected by the *bak1–5* mutation and required for postinvasive resistance against *Ab* (Fig. 7). Our further analyses revealed that this pathogen activates the expression of distinct defense-related genes, including *AED1*, *BGL2/PR2*, *GLIP1*, and *RLP23* (Supplementary Table S1 and Fig. 6). Notably, expressions of these genes were induced following *Ab* invasion, and these were canceled in *bak1–5* mutants (Fig. 6). Therefore, we suggest that *Arabidopsis* deploys a PRR system to sense the invasion of *Ab* and subsequently activate antifungal defense pathways that are uncoupled from the Trp-metabolism (Fig. 7).

Notably, it has been reported that the *glip1* mutant plants exhibit enhanced susceptibility to *Ab*^33^. The recombinant GLIP1 protein exhibits antimicrobial activity that disrupts the *Ab* spores and hyphae^33^ and triggers systemic acquired resistance against bacterial pathogens (e.g., *Erwinia carotovora* and *Pseudomonas syringae*) as well as *Ab*^34^. Thus, the enhanced susceptibility of *Arabidopsis* to *Ab* in the presence of *bak1–5* might be partially caused by the reduced expression of *GLIP1*.

It remains unclear how *Arabidopsis* plant recognizes pathogen invasion and then activates Trp-related metabolite accumulation as key immune responses in postinvasive resistance. Because *PAD3* and *CYP71A12* were commonly induced by the invasion of diverse fungal pathogens such as *Ab* and *Ctro*, we suggest that *Arabidopsis* probably recognizes the cell damage that is commonly caused by pathogen invasion and then activates Trp-related metabolism. Further studies are needed to explore a recognition mechanism of pathogen invasion that activates these secondary metabolic pathways for antifungal defense.

## Materials and methods

### Fungal materials

*C*. *tropicale* (*Ctro*) (formerly *Colletotrichum gleosporioides* S9275) was provided by Shigenobu Yoshida (National Institute for Agro-Environmental Sciences, Japan); and *A*. *brassicicola* (*Ab*) strain Ryo-1 was provided by Akira Tohyama. Cultures of *Ab* were maintained on 3.9% (w/v) potato dextrose agar medium (PDA; Nissui Pharmaceutical Co., Ltd., Tokyo, Japan) at 24 °C in the dark. *Ctro* was cultured on 2.5% (w/v) PDA (Difco, Detroit, MI, USA) at 24 °C under a cycle of 16 h black light (FS20S/BLB 20 W; Toshiba, Tokyo, Japan) illumination and 8 h dark.

### *Arabidopsis* lines and growth conditions

The *A*. *thaliana* accession Col-0 was used as the WT plant. The mutants *pen2-1*, *pen2-2*^2^, *pad3-1*^40^, *cyp71A12*, *cyp71A12* *cyp71A13*^41^, *cyp82C2*^14^, *bak1–4*^19^, *bak1–5*^25^, *cyp79B2 cyp79B3*^11^, *pen2 pad3*^10^, *pen2 cyp82C2*^15^, *pen2 cyp71A12*^15^, *pen2 cyp71A12 cyp71A13*^15^, *cyp71A12 cyp71A13 myb34 myb51 myb122*^15^, *pen2 bak1–4*^30^ and *pen2 bak1–5* (generated in this study) were used in this study. *Arabidopsis* seeds were sown on rockwool (Grodan; http://www.grodan.com) and kept at 4 °C in the dark for 2 days, and later grown at 25 °C with a cycle of 16 h light and 8 h dark in Hoagland medium.

### Pathogen inoculation, lesion development analysis and trypan blue viability staining assay

For spray inoculation assays of *Ab*, 5 × 10^5^ conidia/mL of conidial suspension was spray-inoculated on 4–5-week-old plants. For drop-inoculation, 5 μL of conidial suspensions of *Ab* (1 × 10^5^ conidia/mL) were placed onto each leaf. The inoculated plants were kept at 25 °C with a cycle of 16 h light and 8 h dark and maintained at 100% relative humidity. For analysis of lesion development following the inoculation of *Ab*, four drops of 5 μL conidial suspension of each pathogen were drop-inoculated on each leaf, and 24–50 lesions were evaluated in each experiment. The developed lesions were quantified using ImageJ image analysis software (http://imagej.net) and relative values to WT (Col-0) plants were calculated. To measure lesion areas, yellowish areas were included as lesions. Trypan blue staining was conducted according to the method previously described^42^. For the trypan blue assay, at least 50 lesions were investigated in each experiment. The *Ab* invasion ratio (%) was calculated by using the following formula: *Ab* invasion ratio (%) = (number of germinating conidia that developed invasive hyphae / number of germinating conidia) × 100.

### Generation of mutant plants

The generation of *pen2 bak1–5* line used in this study was generated by crossing the *pen2–1* mutant with *bak1–5* plants. The genotype was checked with the corresponding specific primers for the derived cleaved-amplified polymorphic sequence (dCAPS) markers using dCAPS Finder 2.0 (http://helix.wustl.edu/dcaps/dcaps.html), and the PCR products (WT or mutant types) were cleaved with appropriate restriction enzymes (Supplementary Table S2).

### RT–qPCR analysis

Seven *Arabidopsis* leaves inoculated with *Ab* (5 × 10^5^ conidia/mL) were collected from each of seven different plants of either WT Col-0 or mutant plants at corresponding time points. Total RNA was extracted using PureLink (TRIzol plus RNA purification kits, Life Technologies/Thermo Fisher Scientific, Waltham, MA, USA) and treated with DNase (RQ1 RNase-free DNase; Promega, Madison, WI, USA; http://www.promega.com) to remove DNA contamination. Takara Prime Script RT kits (Takara Bio Inc., Shiga, Japan; http://www.takara-bio.com) was used for the cDNA synthesis. Takara TB Green Premix Ex Taq I was used for RT–qPCR, performed using the primers listed in Supplementary Table S2. Arabidopsis *UBC21* (At5g25760) was used as an internal control for normalizing the level of cDNA^43^. RT–qPCR analysis was performed using a Thermal Cycler Dice Real Time System TP800 (Takara). The expression levels of genes of interest were normalized relative to those of *UBC21*.

### Metabolite analysis

Conidial suspensions (5 × 10^5^ conidia/mL) of *Ab* were spray-inoculated onto 4–5-week-old plants and kept at 100% relative humidity. Leaf samples (100–200 mg fresh weight) were collected at corresponding time points and frozen immediately in liquid nitrogen. The plant extracts containing Trp derivatives were extracted using DMSO and metabolite analyses were performed as described^4,15^.

### Microarray analysis

*Ab* conidial suspensions (5 × 10^5^ conidia/mL) were spray-inoculated onto 4–5-week-old plants of the *pen2* and *pen2 bak1–5* mutants. For each sample, five leaves were collected at 24 hpi and frozen immediately in liquid nitrogen. In total, eight samples (four biological replicates each of the mock and *Ab*-treated samples) were used for RNA extraction. Total RNA was extracted using Plant RNA Isolation Mini kits (Agilent Technologies., Santa Clara, CA, USA). Aliquots of 200 ng of total RNA were used to prepare Cy3-labeled cRNA using Agilent Low Input Quick Amp labeling kits. The labeled samples were hybridized onto an Agilent *Arabidopsis thaliana* microarray (ver. 4.0; 4 × 44 K format). After hybridization and washing, the arrays were scanned using an Agilent microarray scanner (G2565BA). The images were analyzed using Agilent Feature Extraction software (ver. 10.7.3.1), and further analysis was performed using Agilent GeneSpring GX12.1 software. Signal normalization was based on the expression ratio of *pen2 bak1–5* to *pen2*. Differentially upregulated genes were defined as having a greater than 2.5-fold increase in expression, and differentially downregulated genes were defined as having at least a 0.4-fold decrease in expression. Microarray data have been deposited in the NCBI Gene Expression Omnibus (GEO) database GSE 124921.

## Supplementary Information

Supplementary Information.
