# Supplementary material for: Tryptophan-derived metabolites and BAK1 separately contribute to Arabidopsis postinvasive immunity against Alternaria brassicicola
Source: Sci Rep. 2021 Jan 15;11:1488. doi: 10.1038/s41598-020-79562-x (PMC7810738; doi:10.1038/s41598-020-79562-x)
Supplement: Supplementary file 1 — Supplementary Information. [file 41598_2020_79562_MOESM1_ESM.pdf]

Supplemental information

Tryptophan-derived metabolites and BRASSINOSTEROID INSENSITIVE 1-ASSOCIATED RECEPTOR KINASE 1 separately contribute to *Arabidopsis* postinvasive immunity against *Alternaria brassicicola*

Ayumi Kosaka[1], Marta Pastorczyk[2], Mariola Piślewska-Bednarek[2], Takumi Nishiuchi[3], Erika Ono[1], Haruka Suemoto[1], Atsushi Ishikawa[4], Henning Frerigmann[5], Masanori Kaido[1], Kazuyuki Mise[1], Paweł Bednarek[2], and Yoshitaka Takano[1,*]

[1] Laboratory of Plant Pathology, Graduate School of Agriculture, Kyoto University, Kyoto 606-8502, Japan
[2] Institute of Bioorganic Chemistry, Polish Academy of Sciences, Noskowskiego 12/14, 61-704, Poznan, Poland
[3]Advanced Science Research Center, Kanazawa University, Kanazawa, Japan
[4] Department of Bioscience and Biotechnology, Fukui Prefectural University, Fukui, 910-1195, Japan.
[5] Max Planck Institute for Plant Breeding Research, Carl-von-Linne-Weg 10, D-50829 Cologne, Germany

*Corresponding author (takano.yoshitaka.2x@kyoto-u.ac.jp)

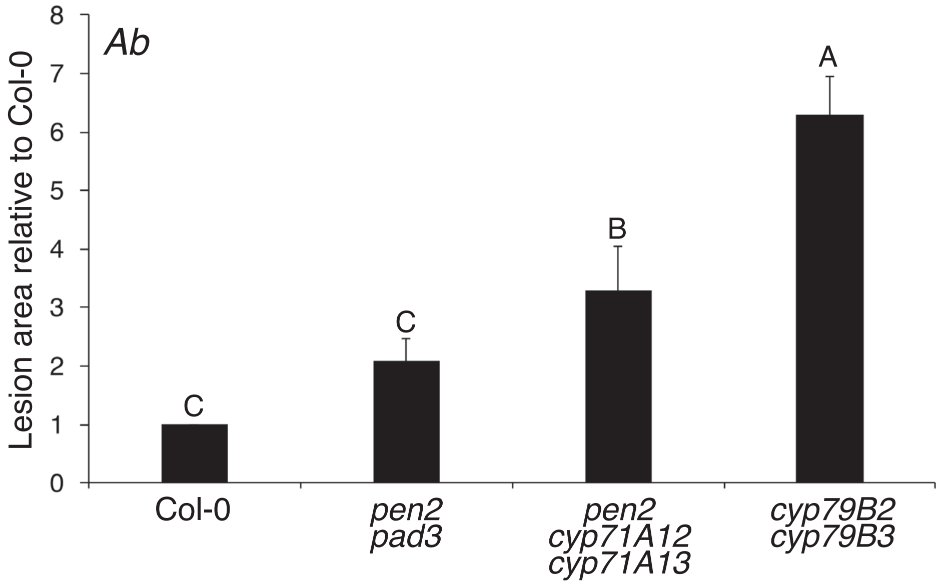

Supplementary Fig. S1. The *cyp79B2 cyp79B3* mutant was more susceptible to *Ab* than was the *pen2 cyp71A12 cyp71A13* mutant. Quantification of lesion development in the *Ab*-inoculated leaves of the tested Arabidopsis lines at an early time point (3 dpi). Conidial suspensions of *Ab* (1 x 10^5 conidia/mL) were drop-inoculated onto true leaves of 4–5-week-old plants. At 3 dpi, lesion areas were measured, and then the relative values to those in Col-0 (WT plants) were calculated. The means and SDs were derived from three independent experiments. The statistical significance of differences between means was determined by Tukey's honestly significant difference (HSD) test. Means not sharing the same letter are significantly different ($P < 0.05$).

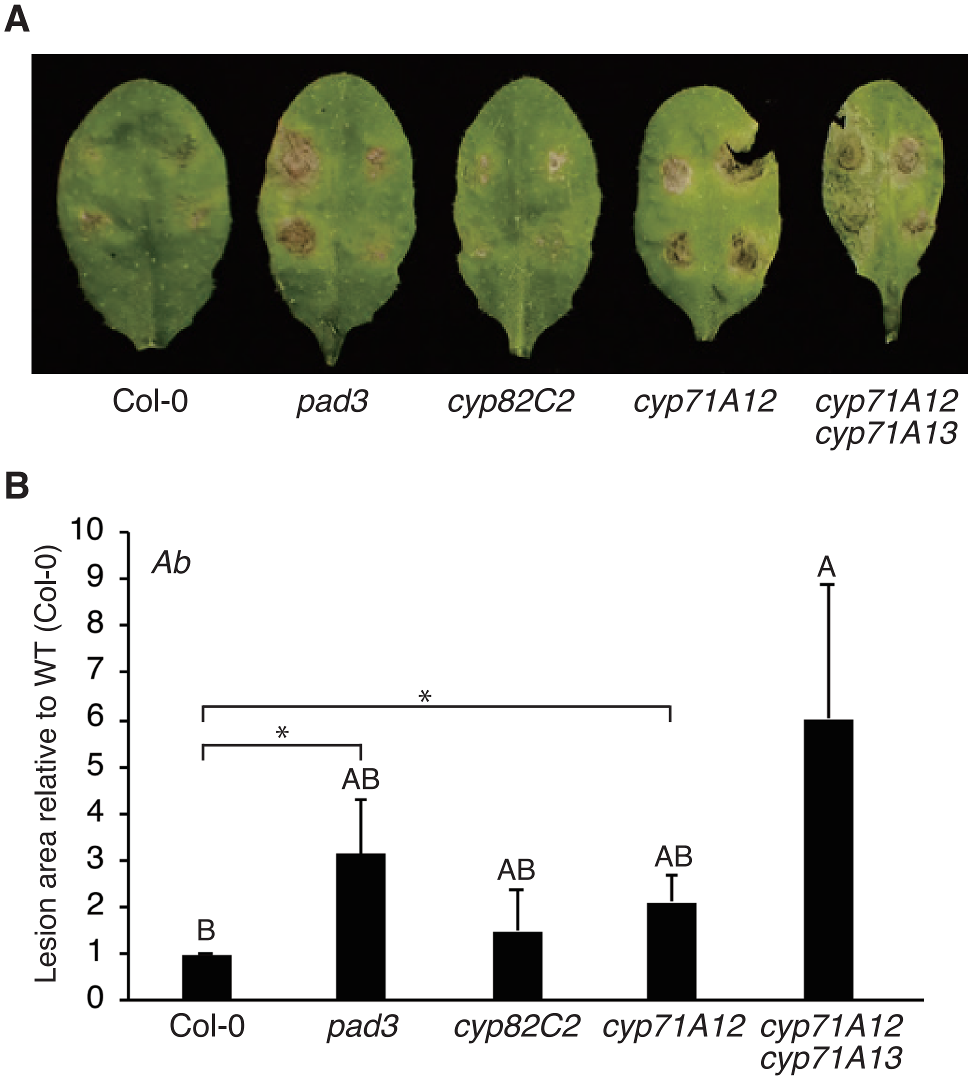

Supplementary Fig. S2. (A) Lesion development caused by *Ab* on *Arabidopsis* mutant plants. Conidial suspensions (1 x 10^5 conidia/mL) of *Ab* were drop-inoculated onto the tested mutant lines. This photograph was taken at 3 dpi. **(B)** Quantification of lesion development. Conidial suspensions of *Ab* were drop-inoculated onto tested plants. At 3 dpi, lesion areas were measured and the relative values to Col-0 (WT plants) were calculated. Means and standard deviations (SDs) were calculated from three independent experiments. The statistical significance of differences between means was determined by Tukey's honestly significant difference (HSD) test. Means not sharing the same letter are significantly different ($P < 0.05$). In *pad3*, *cyp82C2* and *cyp71A12* mutants, one-tailed Student's *t*-test was also performed to compare with Col-0 (WT), and asterisks indicate the significant differences (*$P < 0.05$).

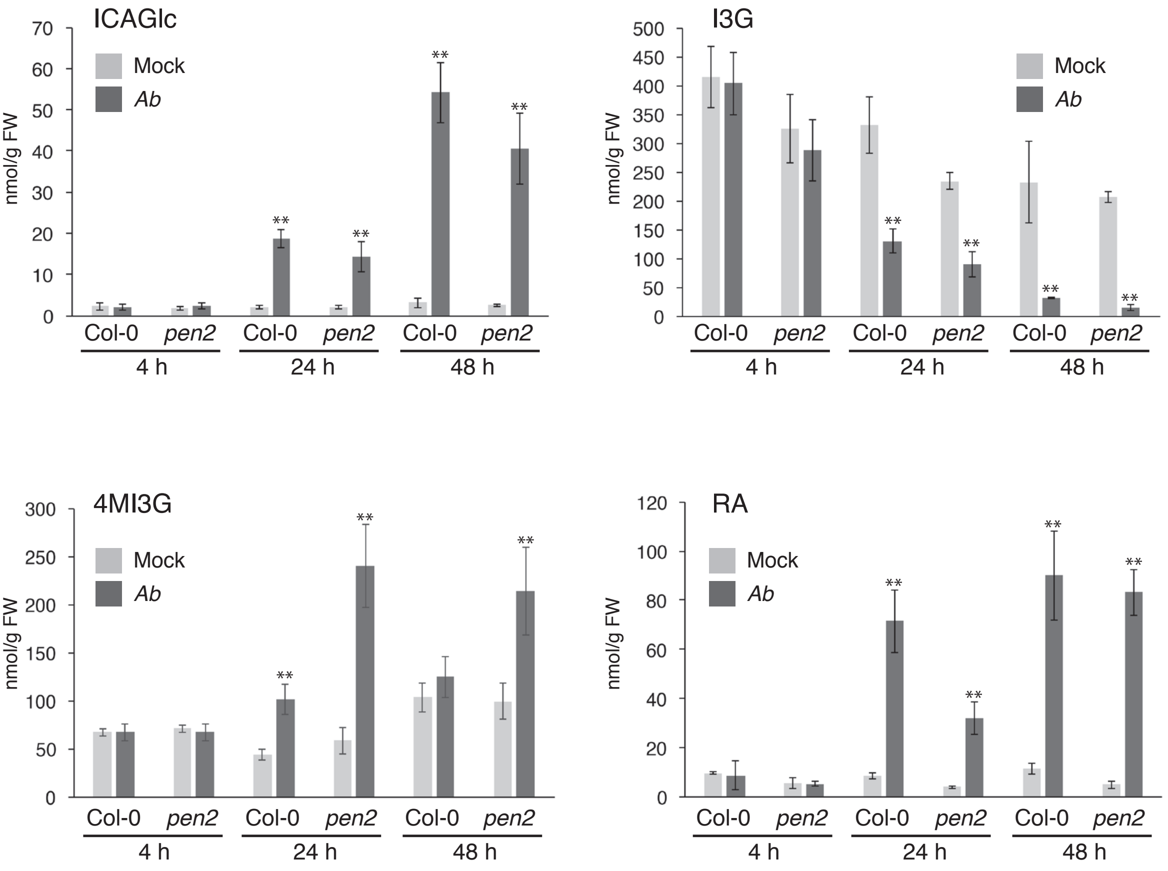

Supplementary Fig. S3. The analysis of ICAGlc, I3G, 4MI3G, and RA accumulation in WT (Col-0 plants) and the *pen2* mutant inoculated with *Ab*. Conidial suspensions (5 x 10^5 conidia/mL) of *Ab* were spray-inoculated onto WT and *pen2* plants. As a control, water was sprayed as a mock treatment. The samples were collected at 4, 24, and 48 hpi. The accumulation of indole-3-carboxylic acid glucose ester (ICAGlc), indole-3-ylmethylglucosinolate (I3G), 4-methoxyindol-3-ylmethylglucosinolate (4MI3G), and raphanusamic acid (RA) were determined. The means of metabolites (nmol/g FW) and SDs from four biological independent samples are shown in the graph. The statistical analysis between mock- and *Ab*-treated samples at each time point was conducted using two-tailed Student's *t* tests (***P* < 0.01).

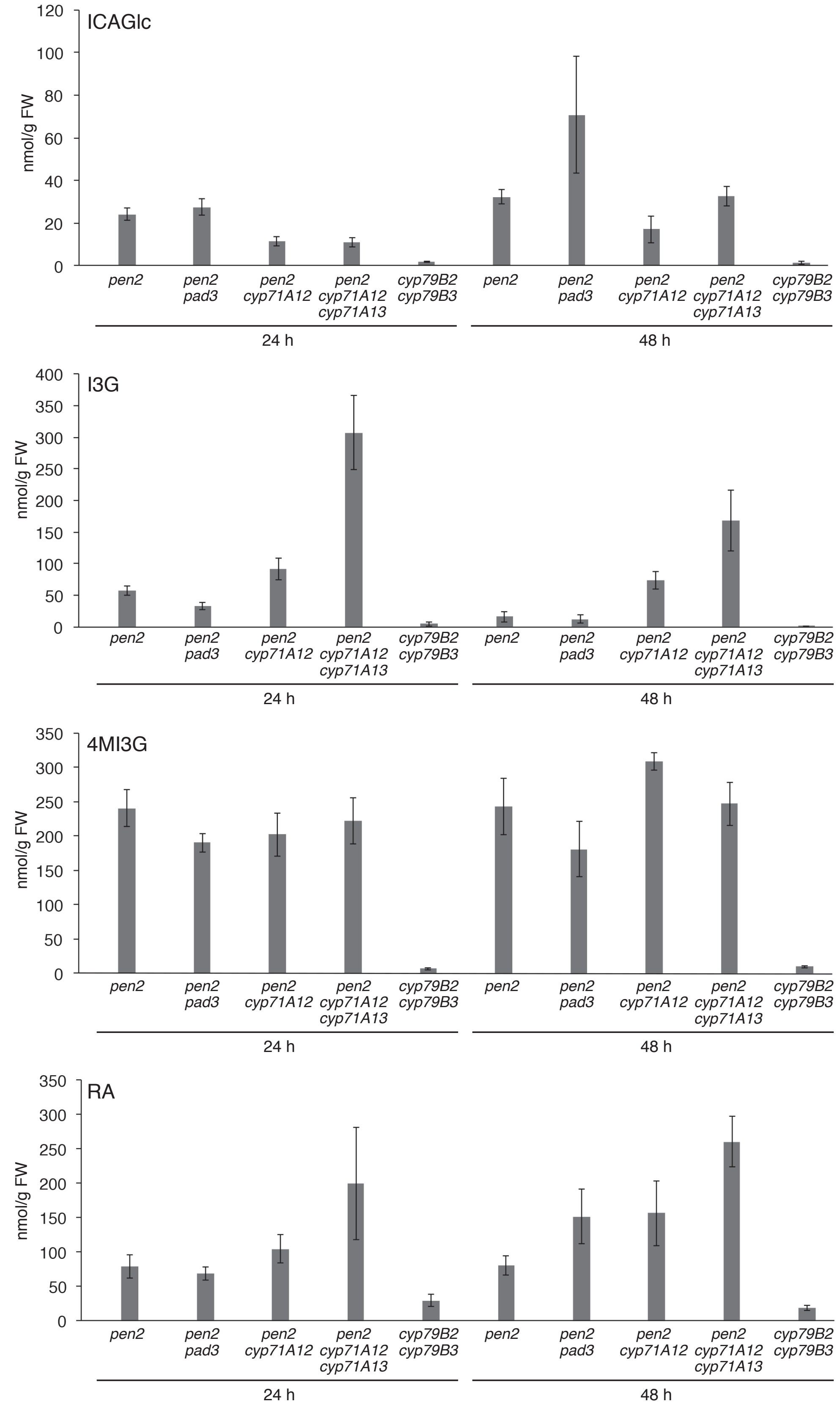

Supplementary Fig. S4. The analysis of ICAGlc, I3G, 4MI3G, and RA accumulation in a series of mutants defective in Trp metabolism. Conidial suspensions (5 x 10^5 conidia/mL) of *Ab* were spray-inoculated onto the tested mutant plants. The samples were collected at 24 and 48 hpi. The accumulations of ICAGlc, I3G, 4MI3G, and RA were determined. The means of metabolites (in nmol/g FW) and SDs from four biological independent samples are shown in the graph.

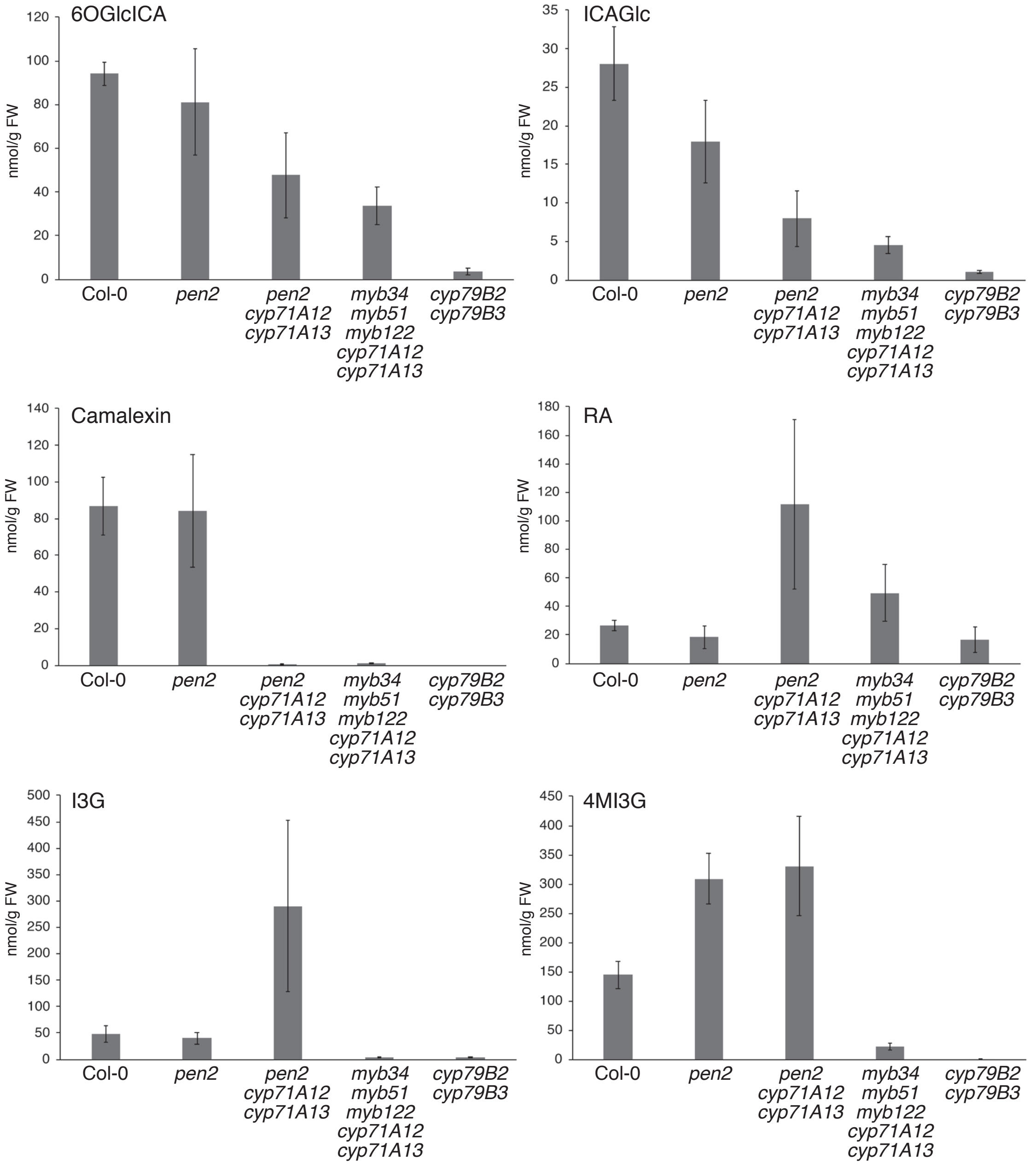

Supplementary Fig. S5. The analysis of Trp-derived metabolite accumulation in the *myb34 myb51 myb122 cyp71A12 cyp71A13* plants upon *Ab* inoculation. Conidial suspensions (5 x 10^5 conidia/mL) of *Ab* were spray-inoculated onto the tested mutant plants. The samples were collected at 24 hpi. The means of metabolites (in nmol/g FW) and SDs from four biological independent samples are shown in the graph.

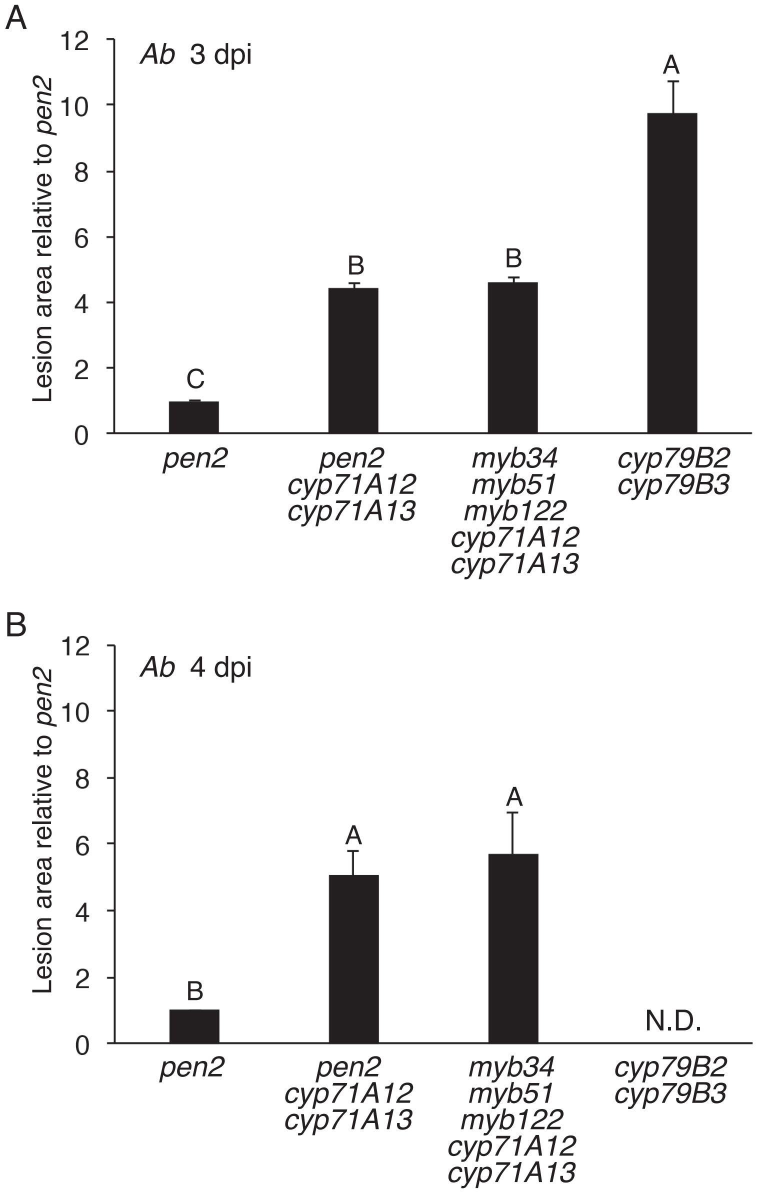

Supplementary Fig. S6. Higher susceptibility to *Ab* in *cyp79B2 cyp79B3* than in *pen2 cyp71A12 cyp71A13* mutants is not caused by the accumulation of PEN2-independent indole-glucosinolates. Conidial suspensions of *Ab* (1 x 10^5 conidia/mL) were drop-inoculated onto true leaves of 4–5-week-old plants. At 3 dpi (A) and 4 dpi (B), lesion areas were measured, and relative values to the *pen2* mutant were calculated. The means and SDs were derived from three independent experiments. The statistical significance of differences between means was determined by Tukey's honestly significant difference (HSD) test. Means not sharing the same letter are significantly different ($P < 0.05$). ND; not determined.

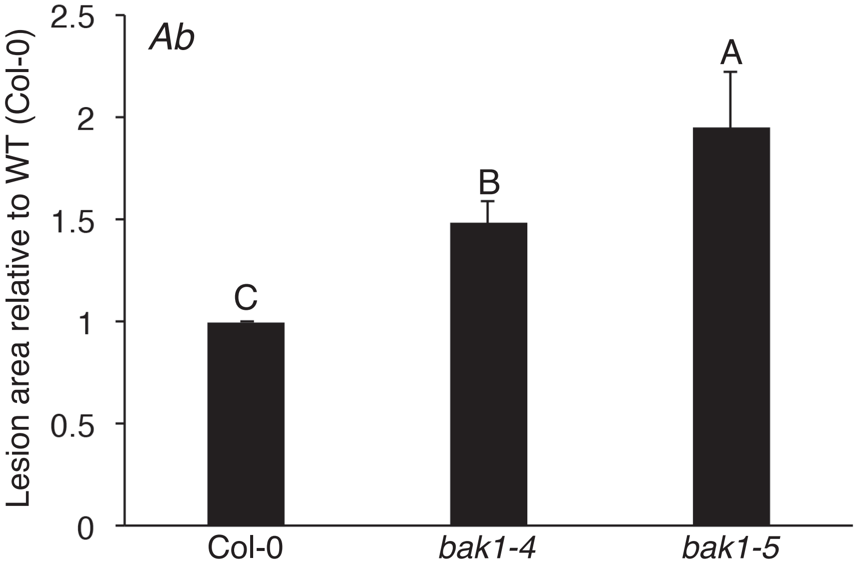

Supplementary Fig. S7. The *bak1* mutants showed enhanced susceptibility toward *Ab* compared with the WT plants (Col-0). Conidial suspensions of *Ab* (1 x 10^5 conidia/mL) were drop-inoculated onto the tested plants. At 4 dpi, lesion development relative to WT plants was determined. The means and SDs were derived from three independent experiments. The statistical significance between the means was determined by Tukey's honestly significant difference (HSD) test. Means not sharing the same letters are significantly different ($P < 0.05$).

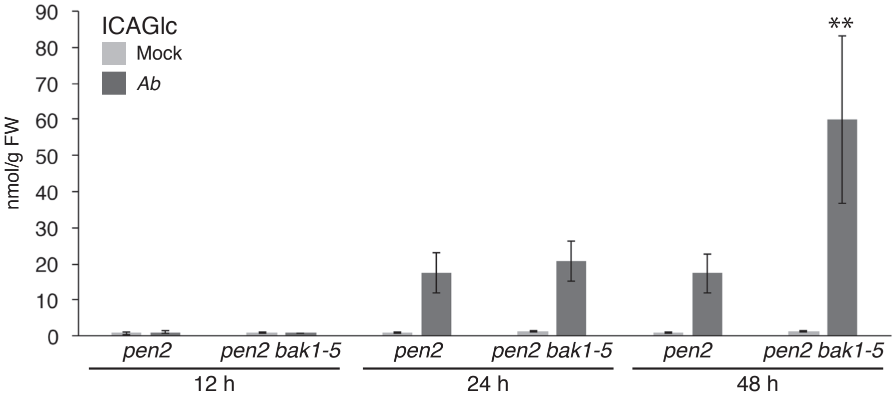

Supplementary Fig. S8. The analysis of ICAGlc accumulation in both *pen2* and *pen2 bak1–5* mutants. Conidial suspensions (5 x 10^5 conidia/mL) of *Ab* were spray-inoculated onto the tested plants. As a control, water was sprayed as a mock treatment. The samples were collected at 12, 24, and 48 hpi. The means of metabolites (nmol/g FW) and SDs from four biological independent samples are shown in the graph. Comparisons between *pen2* and *pen2 bak1–5* at each time point were conducted using two-tailed Student's *t* tests (**$P < 0.01$).

Supplementary Table S1. List of differentially regulated *Arabidopsis* genes in *pen2 bak1–5* plants after *Ab* invasion at 24 hpi

Locus	Description	*pen2 bak1-5/pen2* [a]
Down-regulated in *pen2 bak1-5*		
At5g52740	copper transport family protein	0.20
At4g04500	cysteine-rich receptor-like protein kinase 37 (*CRK37*)	0.21
At2g26400	acireductone dioxygenase 3 (*ARD3*)	0.21
At4g23150	cysteine-rich receptor-like protein kinase 7 (*CRK7*)	0.29
At3g57260	beta 1,3-glucanase 2 (*BGL2*)	0.30
At5g55450	bifunctional inhibitor/lipid-transfer protein/seed storage 2S albumin-like protein	0.31
At5g10760	aspartyl protease family protein (*AED1*)	0.33
At2g14580	pathogenesis-related protein 1 (*PRB1*)	0.34
At2g32680	receptor like protein 23 (*RLP23*)	0.36
At5g40990	GDSL lipase 1 (*GLIP1*)	0.37
At3g28510	AAA-type ATPase family protein	0.39
Up-regulated in *pen2 bak1-5*		
At1g29660	GDSL esterase/lipase	25.80
At5g44420	ethylene- and jasmonate-responsive plant defensin (*PDF1.2*)	4.39
At1g79400	cation/H(+) antiporter 2 (*CHX2*)	2.58

[a]Up-regulated genes were determined by a greater than 2.5-fold change of normalized signals in their expression ratio (*pen2 bak1-5* vs *pen2*).
Down-regulated genes were determined by a smaller than 0.4-fold change of normalized signals in their expression ratio (*pen2 bak1-5* vs *pen2*).
The values are the average ratios of four arrays.

Supplementary Table S2. List of primers used in this study

Primers used for RT-qPCR

Gene name	Forward primer sequence (5'-3')	Reverse primer sequence (5'-3')
CYP71A12 (AT2G30750)	CATTCCCTAAGCCTTCGGTAC	CTTGGAGTTTCTTCATAACA
CYP82C2 (AT4G31970)	CATTTGGTTCGGGAAGAAGA	AGCCAGGGCTCTCAGTCATA
PAD3 (AT3G26830)	TGCTCCCAAGACAGACAATG	GTTTTGGATCACGACCCATC
UBC21 (AT5G25760)	CTGCGACTCAGGGAATCTTCTAA	TTGTGCCATTGAATTGAACCC
AED1 (AT5G10760)	GCGTATACAGTATCGTTTACGGCG	GGTAGTGAGAGTTTGCCAGGGCC
BGL2 (AT3G57260)	TGGATCACCGAGAAGGCCAGGG	GCCCACAAGTCTCTAAGGATTAG
GLIP1 (AT5G40990)	GAGCTGATTTGGAGCGGACCTACC	CGAGTGATATATATCGCTCGCG
RLP23 (AT2G32680)	GGAGTGGCTTGTCAAGATAATTGG	CCCAATTTTATCCTCATTTGCCCG

Primers used for genotyping

Mutant name	Forward primer sequence (5'-3')	Reverse primer sequence (5'-3')	Enzyme
pen2-1	TCAGGTAAATCAGTTCGAATCAAGAAC	TGAGGAAACCTGTTGGAGAAAGGATC	BamHI
bak1-5	AAGAGGGCTTGCGTATTTACATGATCAGT	GAGGCGAGCAAGATCAAAAG	RsaI

Supplementary Table S3. List of *Arabidopsis thaliana* lines used in this study

Arabidopsis thaliana lines	Reference
Col-0 (WT)	
pen2-1	Lipka *et al.,* 2005
pen2-2	Lipka *et al.,* 2005
pad3-1	Glazebrook and Ausubel, 1994
cyp71A12	Müller *et al.,* 2015
cyp71A12	Rajniak *et al.,* 2015
bak1-4	Chinchilla *et al.*, 2004
bak1-5	Schwessinger *et al,* 2011
cerk1-2	Miya *et al.,* 2007
cyp71A12 cyp71A13	Müller *et al.,* 2015
cyp79B2 cyp79B3	Zhao *et al.,* 2002
pen2 pad3	Bednarek *et al.,* 2009
pen2 cyp82C2	Pastorczyk *et al.,* 2019
pen2 cyp71A12	Pastorczyk *et al.,* 2019
pen2 bak1-4	Takahashi *et al*., 2016
pen2 bak1-5	In this study
pen2 cyp71A12 cyp71A13	Pastorczyk *et al.,* 2019
cyp71A12 cyp71A13 myb34 myb51 myb122	Pastorczyk *et al.,* 2019
